# The Switch from NF-YAl to NF-YAs Isoform Impairs Myotubes Formation

**DOI:** 10.3390/cells9030789

**Published:** 2020-03-24

**Authors:** Debora Libetti, Andrea Bernardini, Sarah Sertic, Graziella Messina, Diletta Dolfini, Roberto Mantovani

**Affiliations:** Dipartimento di Bioscienze, Università degli Studi di Milano, Via Celoria 26, 20133 Milano, Italy; debora.libetti@unimi.it (D.L.); andrea.bernardini@unimi.it (A.B.); sarah.sertic@unimi.it (S.S.); graziella.messina@unimi.it (G.M.); diletta.dolfini@unimi.it (D.D.)

**Keywords:** splicing isoforms, CRISPR-Cas9, exon deletion, NF-Y, muscle differentiation, C2C12 cells

## Abstract

NF-YA, the regulatory subunit of the trimeric transcription factor (TF) NF-Y, is regulated by alternative splicing (AS) generating two major isoforms, “long” (NF-YAl) and “short” (NF-YAs). Muscle cells express NF-YAl. We ablated exon 3 in mouse C2C12 cells by a four-guide CRISPR/Cas9n strategy, obtaining clones expressing exclusively NF-YAs (C2-YAl-KO). C2-YAl-KO cells grow normally, but are unable to differentiate. Myogenin and—to a lesser extent, MyoD— levels are substantially lower in C2-YAl-KO, before and after differentiation. Expression of the fusogenic Myomaker and Myomixer genes, crucial for the early phases of the process, is not induced. Myomaker and Myomixer promoters are bound by MyoD and Myogenin, and Myogenin overexpression induces their expression in C2-YAl-KO. NF-Y inactivation reduces MyoD and Myogenin, but not directly: the Myogenin promoter is CCAAT-less, and the canonical CCAAT of the MyoD promoter is not bound by NF-Y in vivo. We propose that NF-YAl, but not NF-YAs, maintains muscle commitment by indirectly regulating Myogenin and MyoD expression in C2C12 cells. These experiments are the first genetic evidence that the two NF-YA isoforms have functionally distinct roles.

## 1. Introduction

Cell specification and differentiation during development of multicellular organisms is a complex set of events resulting in the formation of organs, whose physiology is maintained by a balance of cell proliferation and differentiation. A paradigmatic example of these phenomena is formation of skeletal muscle. In the case of mammals—mouse in particular—the process begins at early developmental stages, proceeding through embryonic, fetal and adult stages [1,2]. Sequence-specific transcription factors—TFs—play a central role in specifying the identities of myoblasts, their migration to different body locations, organization and the capacity to self-renew and differentiate into myotubes. These properties are key to guarantee maintenance and functionality of the different muscles throughout the lifespan of the organism, including repair after injury in adult life. A set of four key TFs —MyoD, Myf5, Myogenin, MRF4, termed myogenic regulatory factors (MRFs)—have been identified and thoroughly studied by genetic and biochemical means for their capacity to specify myoblasts identity [3,4]. During development, PAX3/7 are located upstream of MRFs [5]; downstream are many TFs, such as the MADS box MEF2A/C/D [6,7], the bHLH ID1/3 [8,9,10] and SNAI1 [11], the HOX SIX1/4/5 [12,13,14,15], STAT3 [16], NFIX [17,18] and the ZNF KLF2/4/5 [19,20]. Unlike MRFs, most of these TFs are not expressed predominantly in muscle cells and are equally important for development and differentiation of other tissues and organs [21,22,23,24,25].

NF-Y is an evolutionarily conserved heterotrimer formed by the sequence-specific NF-YA and the Histone Fold Domain—HFD—NF-YB/NF-YC [26]. The sequence recognized by NF-Y is the CCAAT box, which plays an important role in the activation of 25%–30% of mammalian genes. NF-Y has been classified as ”pioneer” TF, in mammals and plants [27,28,29,30,31]. NF-YA is the regulatory subunit; it is alternatively spliced, generating two major isoforms “short” (NF-YAs) and “long” (NF-YAl), differing in 28 amino acids coded by exon 3 [32]. This stretch is located at the N-terminus of the protein, in the Gln-rich transactivation domain (TAD). NF-YAs and NF-YAl have identical subunits-interactions and DNA-binding properties *in vitro*; ChIP-seq from cells harboring predominantly either one of the two isoforms showed recovery of peaks enriched in CCAAT. The isoforms are expressed at various levels in different tissues and cell lines [32,33]. Importantly, no cell line has been so far described lacking NF-YA—nor the HFDs—and NF-YA inactivation was reported to be fatal to cells [28,34]. NF-YAl is the predominant isoform in muscle C2C12 cells: it is abundant in proliferating cells, but it drops to low levels following terminal differentiation to myotubes, unlike the HFD partners [35,36,37]. Highly reduced NF-YA protein was found in myotubes of adult mice [38]. This suggested that genes up-regulated in the terminal phases of muscle differentiation are either CCAAT-less or not NF-Y-dependent, whereas the trimer activates cell-cycle and growth-promoting genes required during the proliferative state. However, overexpression of NF-YAl led to improved differentiation of C2C12 [39], suggesting that NF-YAl does take part in the differentiation process.

For decades, C2C12 myoblast cells have represented an informative tool to identify genes involved in muscle differentiation [40]. Ablation of the whole NF-YA gene is early embryonic lethal [41], and KO in stable cell lines could not be generated so far. We investigated the role of NF-YAl by genetically ablating exon 3, leading to the production of an intact NF-YAs. We successfully generated homozygous C2C12 lines expressing only NF-YAs and went on to study differentiation properties.

## 2. Materials and Methods

### 2.1. Cell Culture and Proliferation Assay

Mouse myoblast cells (C2C12, ATCC) were cultured in Dulbecco’s modified Eagle’s medium (DMEM) supplemented with 10% Fetal Bovine Serum (FBS, Gibco-Thermo Fisher Scientific), 4 mM l-Glutamine, 100 units/mL penicillin and 100 µg/mL streptomycin (GM, growth medium), in a humidified 5% CO_2_ atmosphere at 37 °C. C2C12 cells differentiation was induced plating cells in DMEM with 2% horse serum (Gibco-Thermo Fisher Scientific), 4 mM l-Glutamine, 100 units/mL penicillin and 100 µg/mL streptomycin (DM, differentiation medium). Proliferation assay was performed by plating 1.5 × 10^5^ cells into a 12-well plate and counting every 24 h for 3 days, using the Trypan Blue dye exclusion test. All data were gathered from at least three independent biological replicates. Multiple comparisons were performed using the One-way ANOVA test.

### 2.2. Derivation of C2-YAl-KO Clones

To delete the exon 3 of NF-YA gene in C2C12 cells, four guide RNAs (gRNAs) were designed to simultaneously target the two flanking introns by using the online tool https://zlab.bio/guide-design-resources. Potential off-target sites were monitored by the online tool https://crispr.cos.uni-heidelberg.de: Table S1 shows the results of such analysis for the four guides. The selected gRNAs had no common off-target sites and were cloned in the two plasmids pX330A_D10A-1x2_ac and pX330A_ D10A-1x2_bd, following the Multiplex CRISPR/Cas9n Assembly System Kit protocol [42]. 1 × 10^6^ C2C12 cells were transfected with 3 μg of the two gRNAs/CRISPR/Cas9n plasmids by electroporation and plated at low density. 72 h after transfection, single clones were picked, expanded and screened.

For DNA extraction, cells from the individual clones were washed with PBS, collected by scraping, lysed in 100 μL ice-cold lysis buffer (40 mM Tris-HCl, 2 mM EDTA, 0.08% SDS, 80 mM NaCl, 0.5 μg/μL Proteinase K) and incubated overnight at 37 °C in agitation. To precipitate DNA, 100 µL of ice-cold 2-propanol and 0.3 M NaAc were added, samples were mixed and centrifuged at 13,000 rpm for 30 min at 4 °C. The pellet was washed with 150 μL of 70% ethanol, centrifuged at 13,000 rpm for 30 min at 4 °C. Supernatant was discarded, the pellet was dried and resuspended in 30 μL H_2_O. The resulting DNAs were then screened for positive exon 3 deletion by PCR.

We screened a total of 335 individual clones and obtained 2 independent homozygously edited clones.

### 2.3. Protein Extraction and Western Blot Analysis

For Whole Cell Extracts preparation, cells were pelleted by centrifugation, resuspended in ice-cold RIPA buffer (10 mM TrisHCl pH 8.0, 1 mM EDTA, 0.5 mM EGTA, 0.1% SDS, 0.1% sodium deoxycholate, 140 mM NaCl, 1% Triton X-100, 1 mM PMSF, Protease inhibitor cocktail) and incubated for 30 min on ice, with occasional shaking. Samples were centrifuged at 13,000 rpm for 10 min at 4°C and the supernatant recovered and quantified using the Bradford protein assay.

20 μg of extracts were loaded on a 4–10% SDS-polyacrylamide gel and analyzed by Western blot using primary antibodies and a peroxidase-conjugate secondary antibody (Sigma-Aldrich). Primary antibodies: anti-NF-YA (G-2, Santa Cruz), anti-NF-YB (GeneSpin), anti-NF-YC (home-made) anti-Vinculin (H-10, Santa Cruz), anti-MyHCs (MF20, DHSB), anti-Myogenin (IF5D, DHSB), anti-MyoD (C-20, Santa Cruz), anti-Myf5 (C-20, Santa Cruz), anti-Pax3 (DHSB), anti-Snai1 (C15D3, Cell Signaling). Western blot experiments were performed on three independent biological replicates.

### 2.4. Reverse Transcriptase PCR and Real-Time PCR

RNA was isolated by the Tri Reagent (Sigma-Aldrich) protocol according to the manufacturer’s instruction. The cDNA was produced starting from 1 μg of total RNA using the MMLV Reverse Transcription Mix (GeneSpin) and used for real-time PCR (SYBR^®^ Green Master Mix, Bio-rad Laboratories) analysis. Real-time PCRs were performed with oligonucleotides designed to amplify 100–200 bp fragments (Table S2). The housekeeping gene Rsp15a was used to normalize expression data. The relative sample enrichment was calculated with the formula 2^–(ΔΔCt)^, where ΔΔCt = [(Ct sample – Ct Rps15a)_x_ − (Ct sample – Ct Rps15a)_y_], x = sample and y = sample control. RT-qPCR analyses were performed on three independent biological replicates. For ChIP experiments, we figured out the percentage of input immunoprecipitated by NF-YB and nc (negative control) antibodies. Results of three independent experiments were represented as Fold change (Fc) between NF-YB sample and nc sample as: %Input NF-YB/%Input nc.

### 2.5. Flow Cytometry Analyses

Cells were harvested by trypsinization and washed in PBS, fixed in ice-cold 70% ethanol and stored at 4 °C at least 24 hours. Cells were then washed with 1% BSA in PBS and resuspended in 500 μL of PI-staining solution (20 μg/mL Propidium Iodide, 10 μg/mL RNaseA, 1X PBS) at room temperature for 30 minutes, light protected. FACS analyses were performed using the BD FACSCantoII, analyzed with FACSDiva software and quantified with FlowJo. A total of 10^4^ events were acquired for each sample. Three independent FACS experiments were performed.

### 2.6. Immunofluorescence

For immunofluorescence analyses, cells were washed three times with PBS and fixed 10 min with ice-cold acetone-methanol (1:1) at room temperature. After three washes, cells were permeabilized with 0.25% Triton X-100 in PBS for 5 min and incubated 1 h with the primary antibody anti-sarcomeric MyHCs (MF20, DHSB) at room temperature. Cells were washed three times, permeabilized 5 min with 0.25% Triton X-100 in PBS and incubated with secondary FITC anti-mouse antibody (1:500, Sigma-Aldrich) plus DAPI (2 μg/mL) for 40 min at room temperature, light protected. The acquisition was performed by using the inverted microscope Leica DMI6000 B. Three independent immunofluorescence experiments were performed.

### 2.7. Overexpression and RNA Interference Experiments

Myogenin overexpression was performed by electroporating 1 × 10^6^ C2C12 cells with 3 µg of plasmid (pEMSV-Empty/pEMSV-Myog) and plating them in DM for 96 h. Cells were then collected and analyzed. Three independent biological replicates were performed.

For small interfering RNA (siRNA)-mediated knockdown of NF-YB [29], 2 × 10^6^ C2C12 cells were transfected by electroporation with 100 nM of NF-YB [29] or scrambled control siRNA (Qiagen, SI01327193) and plated into a 10 cm plate in GM condition. 72 h after transfection, cells were collected by scraping for total protein preparation and RNA extraction. Gene expression was analyzed performing real-time PCR. Two independent biological replicates of siRNA interference were performed.

### 2.8. Chromatin Immunoprecipitation Assay (ChIP)

ChIPs were performed as described previously [43] with the following modifications. Briefly, 2 × 10^7^ cells were crosslinked using 1% formaldehyde for 7 min, the reaction was quenched with 125 mM glycine and cells were collected by scraping. After lysis, nuclei were resuspended in Sonication buffer (50 mM Tris-HCl pH 8, 10 mM EDTA, 0.1% SDS, 0.5% sodium deoxycholate, protease Inhibitor cocktail) and sonicated (Bioruptor, Diagenode) to obtain fragments of approximately 150–300 bp, verified on agarose gel electrophoresis. Samples were centrifuged at 13,000 rpm for 10 min at 4 °C and supernatants recovered and quantified by Bradford assay. One hundred micrograms of chromatin were immunoprecipitated with 5 μg of anti-NF-YB (GeneSpin) and anti-FLAG (Sigma-Aldrich) antibodies. Protein-G beads (KPL) were used for recovery of antibody-bound proteins. Crosslinking was reversed by incubation at 65 °C overnight. Reactions were digested with RNase A and Proteinase K and DNA purified using the DNA purification kit (PCR clean Up, GeneSpin). The DNA was eluted in TE (10 mM Tris-HCl pH 8, 1 mM EDTA) and used in real-time PCR. Three independent biological replicates of ChIP experiments were performed.

## 3. Results

### 3.1. Ablation of NF-YA Exon 3 in C2C12 Cells by a Four Guides CRISPR/Cas9n Strategy

**Figure 1:**
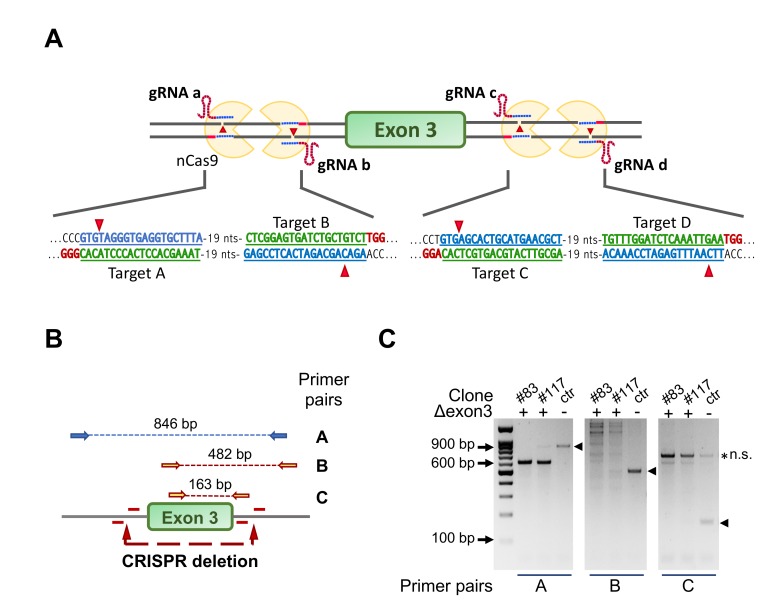
Strategy for ablation of NF-YA exon 3 in C2C12 cells using CRISPR/Cas9n and four gRNAs. (**A**) Gene editing strategy for NF-YA exon 3 deletion using the Cas9-nickase (Cas9n) and four guide RNAs. The targeted sequence by each guide RNA and the deletion sites are shown. Note that Cas9n cuts only the DNA strand that is complementary to and recognized by the gRNA, making necessary the simultaneous presence of two gRNAs/Cas9n complexes to induce a double-strand break (DSB). (**B**) The three primer pairs used to check for positive C2-YAl-KO clones are shown with the specific amplification products highlighted by the dashed lines. (**C**) Example of PCR products run into a 1.2% agarose gel. The expected bands in control cells (ctr) are marked with arrowheads; clones #83 and #117 represent positive C2-YAl-KO clones.

Mouse C2C12 cells mostly express NF-YAl [35,36,37,38]. To study the role of this isoform in maintenance and differentiation of C2C12, we set out a strategy to selectively eliminate exon 3, coding for the 28 extra amino acids present in NF-YAl and absent in NF-YAs. We figured that the use of four guides flanking precisely the exon 3 regions and of the single strand-cutting Cas9-nickase (Cas9n) would minimize off-target effects, which potentially affect the outcome of this technology [44]. Figure 1A shows the design of the four guide oligonucleotides, two couples targeting the 5’ and 3’ intronic DNA flaking exon 3, respectively. The two couples of oligos were first checked for absence of common genomic targets (Table S1) and cloned unpaired in the final pX330A_D10A-1x2_ad and pX330A_D10A-1x2_cb (Figure S1A), also expressing the Cas9n gene. The two plasmids were transfected in growing C2C12 cells by electroporation. Individual clones were isolated, expanded and analyzed by PCR, employing the amplicons shown in Figure 1B. As expected, the strategy was less efficient if compared to the standard use of two guides plus wt Cas9: 335 clones were individually screened and two were positive for correct ablation in homozygosity, as shown in Figure 1C. The results of PCRs of the two positive clones, #83 and #117, show the expected bands for the A, B and C amplicons, absent in the DNA of the parental C2C12 cells. The regions surrounding exon 3 in both clones were then amplified and sequenced: Figure S1B confirms the deletion of coding sequences of exon 3, with somewhat different ends in the two clones. In summary, we successfully ablated NF-YA exon 3, deriving two clones termed C2-YAl-KO. To the best of our knowledge, this is the second system of genome editing describing a clean deletion of an individual exon [45] and the first one employing the Cas9 nickase system coupled with four gRNAs.

### 3.2. Characterization of C2-YAl-KO Cells

**Figure 2:**
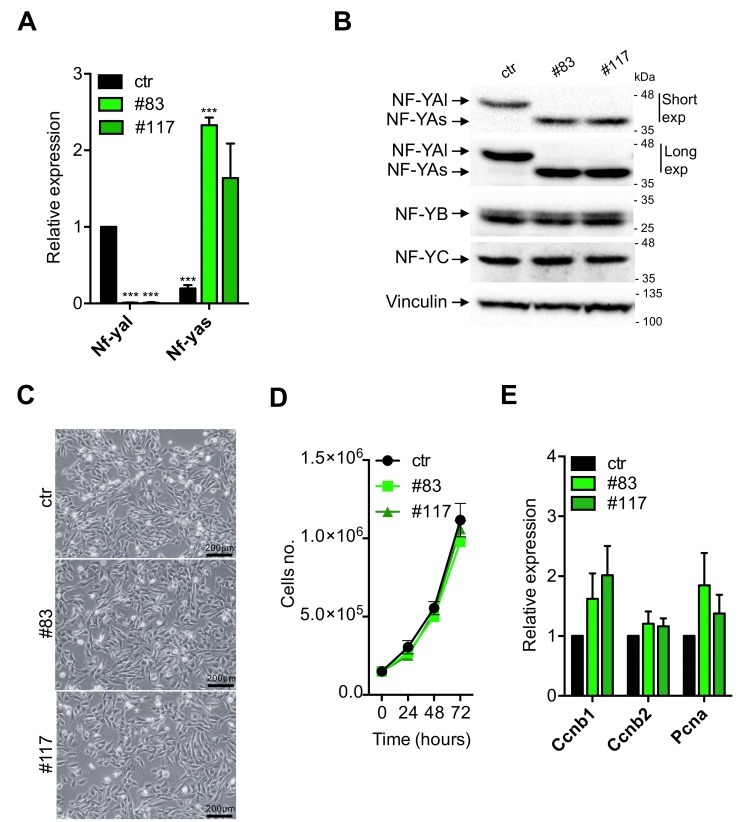
C2-YAl-KO clone characterization. (**A**) Gene expression analysis of NF-YA short and long levels in ctr and C2-YAl-KO clones (#83 and #117) in growth medium (GM) condition. Error bars represent the SD of three independent experiments. P-values were calculated using the one-sample t-test. (**B**) Western blot analysis of NF-Y protein subunits (NF-YA, NF-YB, NF-YC) in ctr cells and C2-YAl-KO clones (#83 and #117) in GM condition. For NF-YA isoforms analysis, short and long exposures are shown. Vinculin was used as loading control. (**C**) Phase contrast analysis of myoblast cells (ctr and C2-YAl-KO clones) morphology in GM condition. Scale bar 200 μm. (**D**) Proliferation assay performed in GM condition counting every 24 h for 3 days using the Trypan Blue dye exclusion test. Error bars represent the SD of three independent experiments. P-values were calculated using the one-way ANOVA test. (**E**) Gene expression analysis of key cell-cycle regulators in ctr and C2-YAl-KO clones (#83 and #117) in GM condition. Error bars represent the SD of three independent experiments. P-values were calculated using the one-sample t-test.

The two C2-YAl-KO clones were characterized first for expression of NF-YA. We performed qRT-PCR analysis with oligos specific for the individual isoforms [46]; Figure 2A shows that the NF-YAl mRNA is absent in the C2-YAl-KO clones. Extracts were prepared and Western blots performed: as expected, the parental C2C12 cells show expression of NF-YAl (Figure 2B). Instead, the clones express uniquely the NF-YAs isoform. We exposed the blots for long times to verify that no NF-YAl is visible in the two KO clones. Note that the levels of the two isoforms in parental cells—NF-YAl—and edited clones—NF-YAs—are essentially identical, as are the levels of NF-YB and NF-YC: since there is an important level of autoregulation among NF-Y subunits [47], this result indicates that HFD subunits are available for trimer formation and DNA-binding in C2C12 and C2-YAl-KO cells. In summary, genetic ablation of exon 3 in C2C12 was effective, leading to generation of clones that express uniquely the short isoform of NF-YA at physiological levels.

Next, we started to analyze the phenotype of the KO clones: they are stable upon repeated cycles of freezing and thawing and their morphology looks apparently similar to the parental C2C12 cells (Figure 2C). In mouse embryonic stem cells, expression of NF-YAs is associated with growth, and NF-YAl to differentiation [43]: in theory, NF-YAs-expressing C2C12 clones could be enhanced in proliferation. Cells were compared for growth under standard conditions: Figure 2D shows that growth curves are similar, with the two edited clones being marginally slower. In FACS analysis, we did notice some differences: a higher number of S-phase and G2/M cells in the two clones (Figure S2, 21% and 28%, with respect to 18% in C2C12). We checked the mRNA levels of PCNA, Cyclin B1/B2: a slight increase of Cyclin B1 and PCNA in the KO clones is observed (Figure 2E); although not statistically significant, this is consistent with the FACS data. The most noticeable difference, however, was the lower number of sub-G1 cells: 6%–7% in the two clones compared to 12% in the parental C2C12 cells (Figure S2): such non cycling cells are possibly undergoing cell death, suggesting that the switch to NF-YAs is not provoking negative effects on cellular vitality, and, if anything, the opposite. In summary, C2-YAl-KO clones expressing NF-YAs have an apparently normal morphology, grow well, but not faster, with the expected partitioning in cell cycle phases, bar slightly elevated G2/M and decreased sub-G1 populations.

### 3.3. C2-YAl-KO Cells Fail to Differentiate and Fuse into Myotubes

**Figure 3:**
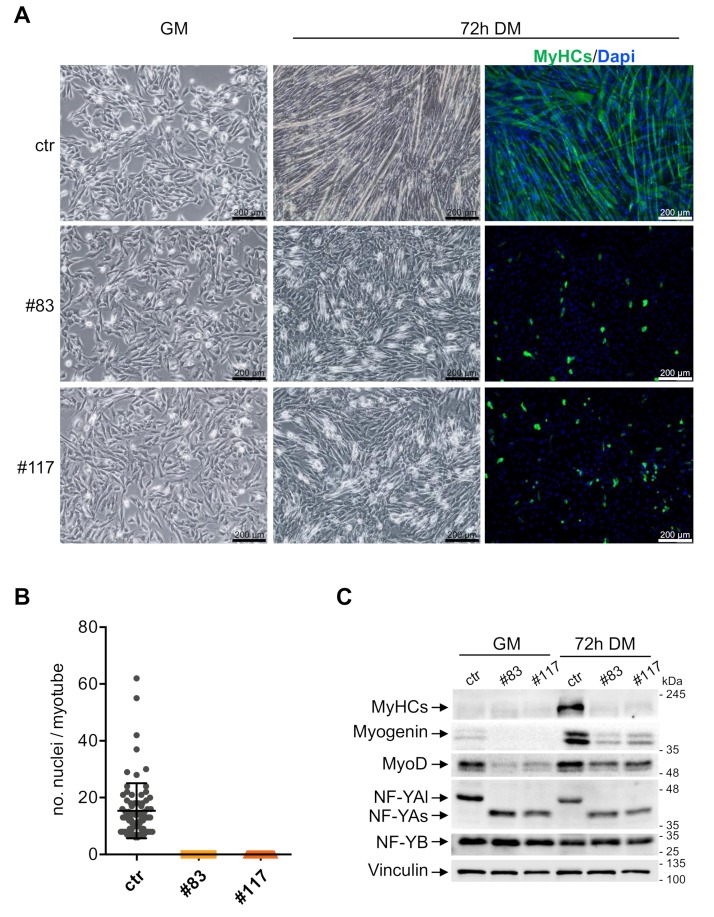
C2-YAl-KO clones fail to differentiate into myotubes. (**A**) Phase contrast analysis of myoblast cells (ctr and C2-YAl-KO clones) before and after 72 h of differentiation (differentiation medium (DM) condition) and immunofluorescence analyses after 72 h of differentiation. Antibody against all sarcomeric MyHCs and DAPI were used. (**B**) Fusion index was calculated as the number of nuclei in each myotube (with three or more nuclei). (**C**) Western blot analysis of key muscle differentiation regulators (MyHCs, MyoD), NF-YA isoforms (NF-YAl, NF-YAs) and NF-YB proteins, before (GM) and after 72 h of differentiation (72 h DM). Vinculin was used as loading control. The experiment was performed three times.

The levels of NF-YAl drop following terminal differentiation of C2C12 cells and myotubes of mouse muscles show low-to-nil levels of NF-YAl [35,36,37,38,39]. To ascertain whether NF-YAs-expressing cells could form myotubes, we switched the parental C2C12 and the two C2-YAl-KO clones at 70%–80% confluence to a differentiation medium. Before and after 72 h, we monitored cell morphology, performed Immunofluorescence experiments and derived whole cell extracts. Figure 3A shows that parental C2C12 form well organized, multinucleated myotubes, as expected (Upper Panels). The average number of nuclei per fiber is 15, in keeping with an efficient process (Figure 3B). On the other hand, the two edited clones showed a dramatic lack of myotubes formation: cells did not fuse; they were disorganized (Figure 3A, lower panels). We reasoned that the process could be simply slower in these cells and prolonged differentiation up to 5 days: this did not lead to formation of myotubes, nor cell fusion in the C2-YAl-KO clones (not shown). Immunofluorescence and Western blot data are consistent: the MyHCs marker is clearly visible in IFs (Figure 3A, right panels) and WB (Figure 3C) in C2C12 cells after differentiation, but not in the two edited clones. Interestingly, the levels of Myogenin and MyoD were substantially lower both in growing cells and at these late stages of differentiation in C2-YAl-KO clones. As previously reported, NF-YAl, in C2C12, and NF-YAs, in the edited clones, are down-regulated after 72 h of differentiation; NF-YB remained unchanged (Figure 3C). In summary, we conclude that terminal differentiation is completely blocked in C2C12 cells expressing NF-YAs instead of NF-YAl.

### 3.4. Expression of TFs in C2-YAl-KO

**Figure 4:**
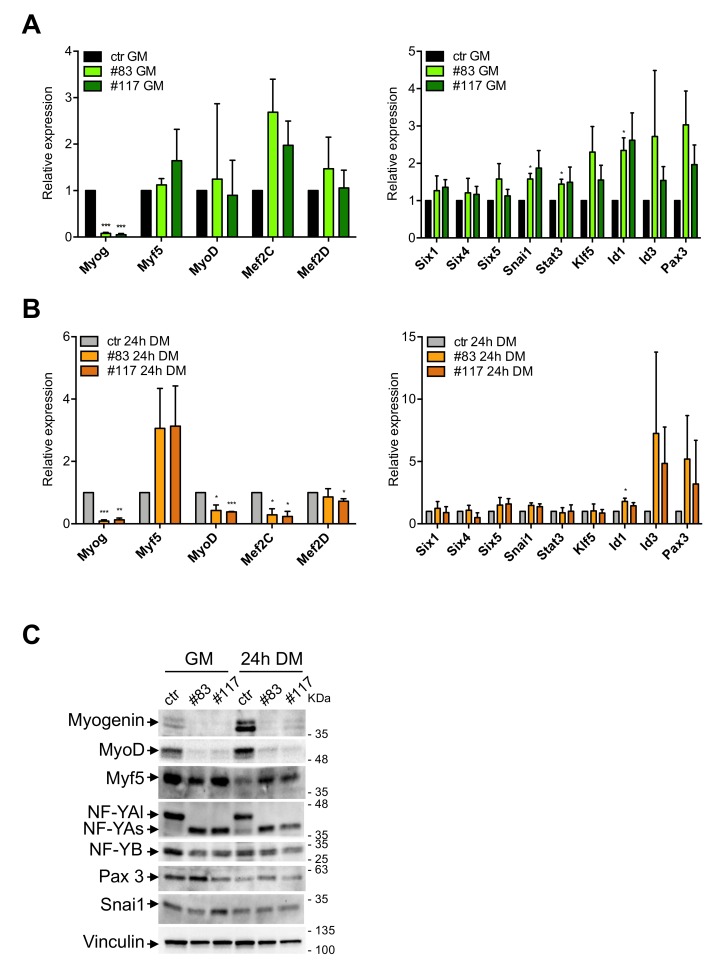
MRFs are downregulated in C2-YAl-KO clones. (**A**) Gene expression analysis of key muscle differentiation regulators (left panel) and other TFs shown to be important for muscle differentiation (right panel) in GM condition. Error bars represent the SD of three independent experiments. P-values were calculated using the one-sample t-test. (**B**) Gene expression analysis of key muscle differentiation regulators (left panel) and other TFs shown to be important for muscle differentiation (right panel) 24 h after differentiation (24 h DM). Error bars represent the SD of three independent experiments. P-values were calculated using the one-sample t-test. (**C**) Western blot analysis of key muscle differentiation regulators (Myogenin, MyoD, Myf5), NF-YA isoforms (NF-YAl, NF-YAs) and NF-YB proteins and other TFs shown to be important for muscle differentiation (Pax3, Snai1), in GM and 24 h DM. Vinculin was used as loading control.

We analyzed expression of MRFs and TFs with a proven role in differentiation, in the parental and in the C2-YAl-KO cells under growing conditions and 24 h after differentiation. Profiling experiments established this as an early time point to detect significant changes in gene expression [48]. Note that most of the TFs analyzed have CCAAT in promoters and some formally shown to be under NF-Y control. First, we verified expression levels of MRFs in parental C2C12 (Figure S3): Myogenin is robustly induced; MyoD is modestly increased; Myf5 is modestly decreased after differentiation; Mef2C, but not Mef2D, is robustly increased. These changes are in agreement with expectations [49]. At the same time, we analyzed other TFs shown to be important for muscle differentiation: Six1/4/5, Snai1, Stat3 and Klf5 are all increased upon C2C12 differentiation, Id1/3 are modestly decreased, Pax3 is unchanged (Figure S3). These results are also in agreement with published data. Having established that our differentiation program runs normally in C2C12 cells, we monitored expression of these genes in the C2-YAl-KO clones. The results are shown in Figure 4A for growing conditions and Figure 4B for differentiation. MRFs show the most conspicuous differences: Myogenin is almost undetectable in growing C2-YAl-KO clones and marginally increased upon differentiation. MyoD basal levels are normal, but induction is reduced upon differentiation, compared to parental C2C12. Myf5 expression is basally similar in the edited clones and higher after differentiation (Figure 4A,B). Mef2C levels are similar in growing conditions, but lower after differentiation: note that the levels are very low basally and differences with parental C2C12 cells are not statistically significant. Mef2D expression is identical in C2C12 and edited clones. As for the other TFs, Six1/4/5, Klf5 and Pax3 show similar expression patterns (Figure 4A,B). Minor changes are observed in growing conditions for Snai1, Stat3 and Id1 (one clone only) and for Id1 (same clone) after differentiation. Finally, Id3 shows somewhat higher levels before and after differentiation, but again, these changes are variable in the three experiments and thus not statistically significant.

To substantiate these results, protein expression of selected TFs was monitored by Western Blot analysis. Figure 4C shows that Myogenin levels are consistent with the mRNA data, being much lower in C2-YAl-KO clones than in parental cells, both in growing cells and after 24 h of differentiation. MyoD is substantially reduced in growing and differentiating clones, compared to parental C2C12. Note that protein levels were far lower than expected based on the mRNA levels, especially under growing conditions: this calls for post-transcriptional control in edited clones. Myf5 protein is downregulated in C2C12 after differentiation, as expected; in edited clones, it shows lower levels in growing cells, but higher after induction. NF-YA and NF-YB show the expected patterns; Pax3 is very modestly increased in C2-YAl-KO clones and Snai1 is unchanged. In summary, C2-YAl-KO cells have substantial differences in MRFs levels with respect to C2C12 cells, both before and after differentiation, whereas the other TFs showed rather minor changes.

### 3.5. Expression of Myomaker and Myomixer Is Activated by Myogenin and It Is Impaired in C2-YAl-KO

**Figure 5:**
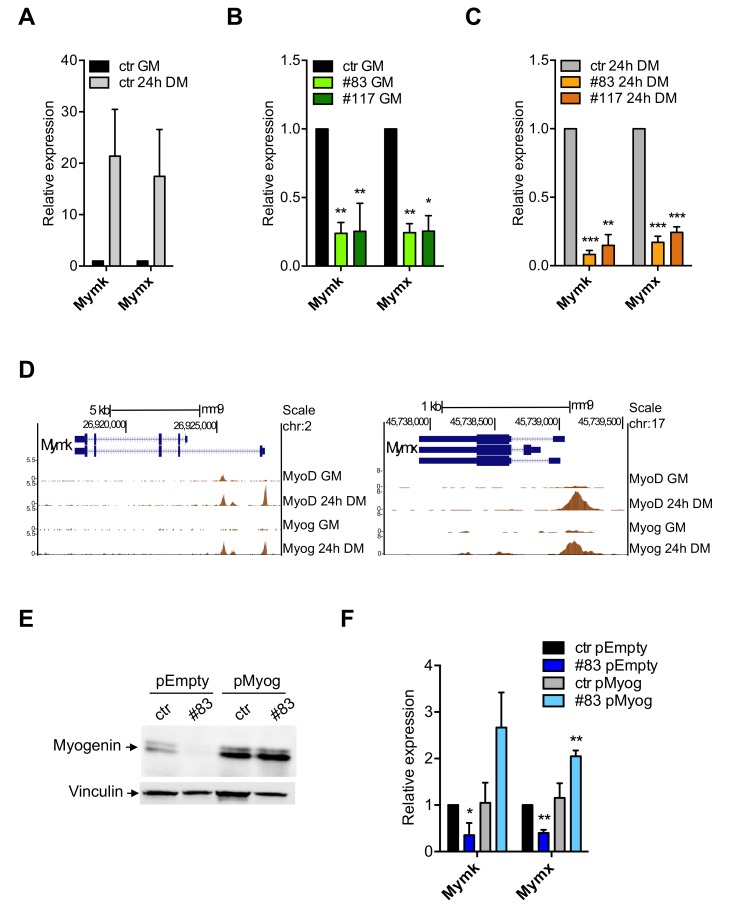
Myogenin directly regulates Myomaker and Myomixer expression. (**A**) Relative expression levels of Myomaker (Mymk) and Myomixer (Mymx) in C2C12 cells before and after 24 h of differentiation (24 h DM). Error bars represent the SD of three independent experiments. P-values were calculated using the one-sample t-test. (**B**,**C**) Relative expression levels of Myomaker (Mymk) and Myomixer (Mymx) in C2C12 cells before (**B**) and after 24 h of differentiation (**C**) in ctr and the two C2-YAl-KO clones. Error bars represent the SD of three independent experiments. P-values were calculated using the one-sample t-test. (**D**) ChIP-seq peaks of MyoD and Myogenin on Mymk and Mymx promoters in GM and after 24 h of differentiation (24 h DM) (UCSC-genome browser available tracks). Vertical viewing range Mymk: min 0, max 5.5. Vertical viewing range Mymx: min 0, max 8. (**E**) Western blot analysis of Myogenin protein levels in C2C12 cells transfected with a control plasmid (pEmpty) and the Myogenin-overexpressing plasmid (pMyog) 96 h after differentiation induction. Vinculin was used as loading control. (**F**) Relative expression levels of Mymk and Mymx in C2C12 Myog-overexpressing cells after 96 h of differentiation. Error bars represent the SD of three independent experiments. *p*-values were calculated using the one-sample t-test.

We were intrigued by the lack of cell fusion of the C2-YAl-KO clones after induction of differentiation. Myomaker—Mymk—and Myomixer—Mymx—are genes induced transcriptionally during muscle terminal differentiation, including in the C2C12 system [50,51]. Specifically, their expression is essential for the process of myocytes fusion [52]. We checked expression by qRT-PCR in parental C2C12 and in the two edited clones 24 h after differentiation. Figure 5A shows a strong induction—20-fold—of both Myomaker and Myomixer in C2C12 cells. C2-YAl-KO have much lower levels in growing cells (Figure 5B) and even more after differentiation (Figure 5C).

The obvious hypothesis was that these genes are under direct NF-Y control. We surveyed their promoter sequences and verified that no bona fide CCAAT box is present, notably within the evolutionary conserved areas: given the specificity of NF-Y CCAAT recognition, we considered unlikely that it acts directly on their expression. Genetic experiments in zebrafish have recently shown that Myomaker and Myomixer are directly activated by Myogenin [53]. We analyzed ENCODE datasets of C2C12 cells and found that Myogenin and MyoD target both promoters. Myomixer has apparently one promoter, Myomaker has two promoters, some 4 kb distant from each other: Figure 5D shows the overlapping peaks of Myogenin and MyoD. Myogenin binds exclusively after 24 h of differentiation, in accordance with its induced expression. One MyoD peak is visible already under growing conditions on Myomaker, and two additional peaks are found at 24 h. Importantly, the regions bound by MyoD and Myogenin in these two promoters are conserved across vertebrates, as shown by PhastCons data in Figure S4A: this corroborates the functional relevance proven in zebrafish [53]. To verify whether Myogenin activates Myomaker and Myomixer, we overexpressed it in parental C2C12 and in one of the C2-YAl-KO clones (#83) and induced to differentiate: Western blot of Figure 5E shows the increased levels of Myogenin compared to cells transfected with an Empty vector control; q-RT-PCR of Figure 5F shows that Myogenin overexpression has negligible effects on expression of the endogenous Myomaker and Myomixer in parental C2C12, but it increases expression of both genes in the C2-YAl-KO cells. Finally, morphological observation of the edited cells shows —incomplete—improvement in differentiation (Figure S4B).

In essence, we find that the marginal levels of Myogenin in C2-YAl-KO cells could result in lack of induction of the Myomaker and Myomixer targeted genes, entailing lack of cell fusion in NF-YAs-expressing clones.

### 3.6. Myogenin and MyoD Are—Indirectly—Regulated by NF-Y

**Figure 6:**
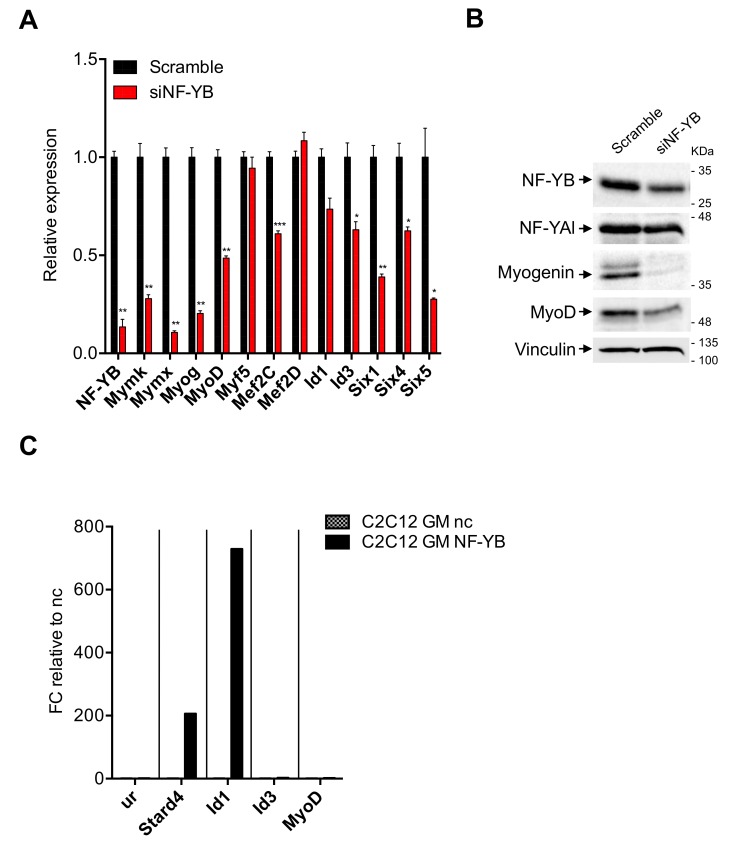
Analysis of NF-Y involvement in muscle specific genes expression. (**A**) Gene expression analysis of NF-YB and key muscle differentiation regulators in C2C12 cells 72 h after NF-YB silencing (siNF-YB) and scrambled siRNA control. Error bars represent the SD of two different RT-qPCR replicates. P-values were calculated using the one-sample t-test. (**B**) Western blot analysis of NF-YB, NF-YA and key muscle differentiation regulators (Myogenin, MyoD) protein levels 72 h after NF-YB silencing (siNF-YB) and the scrambled siRNA control. Vinculin was used as loading control. (**C**) ChIP experiment performed on C2C12 ctr cells in GM condition using NF-YB and negative control (nc) antibodies. The unrelated region (ur) and Stard4 were used as negative and positive control, respectively. Results are represented as the input percentage of each sample normalized to the input percentage of the nc antibody.

The results shown above beg the question as to whether NF-Y directly regulates MRFs. Myogenin and Myf5 promoters do not contain CCAAT boxes, MyoD does [54]. To verify the NF-Y dependence of these genes, we transitorily inactivated NF-Y activity. In our hands, NF-YA inactivations by shRNA or siRNA were rather inefficient in C2C12 cells (not shown). We thus turned to NF-YB by treating C2C12 cells with an siRNA previously shown to be active and very specific, including in profiling experiments [29]. NF-YB is a necessary component of the DNA-binding trimer: this allows us to inhibit CCAAT-binding activity, upon siRNA treatment. Most importantly, unlike NF-YA, NF-YB inactivation does not trigger apoptosis [29,34], making this a suitable choice for long differentiation processes. Figure 6 shows the results of experiment 1, Figure S5 those of experiment 2: in both, RT-qPCR (Figure 6A and Figure S5A) and Western blots (Figure 6B and Figure S5B) show far lower expression of NF-YB in C2C12 cells treated with NF-YB siRNA, with respect to the control siRNA. In mRNA analysis, Myogenin, MyoD and Mef2C, but not Myf5 nor Mef2D, are substantially downregulated upon NF-Y inactivation; Myomaker and Myomixer are also reduced. Six1/4/5 are reduced: for Six4, this in keeping with an NF-Y dependence predicted from previous data on NF-Y binding to a canonical promoter CCAAT [39]. As for Id1 and Id3, they are somewhat reduced, but the results are borderline significant: Id1 in experiment 2 and Id3 in experiment 1. We conclude that NF-Y removal entails a reduction of MRFs, which, in turn, could explain the observed drop of Myomaker and Myomixer. We also show that members of the Six family are NF-Y targets. Analysis of proteins levels in extracts of siRNA-inactivated cells by Western blots confirmed these results: the levels of NF-YB were lower (although not to the extent of the mRNA) and paralleled by somewhat lower levels of NF-YA. Myogenin is substantially decreased and MyoD is also affected, to a lesser extent (Figure 6B and Figure S5B). We conclude that NF-Y regulates the expression of MyoD and Myogenin in C2C12 cells.

The Myogenin promoter is CCAAT-less and was not bound by NF-Y in C2C12 cells [39] and, despite the presence of a canonical CCAAT, the MyoD promoter was also not bound [39]. To understand whether the positive effect of NF-Y on MyoD is direct, we checked the parental C2C12 cells for the presence of NF-Y in ChIP experiments. Three independent experiments are shown in Figure 6C and Figure S5C. The absence of enrichment of NF-Y on MyoD is indeed confirmed, whereas the Stard4 positive control promoter is clearly bound. Equally positive was the promoter of Id1, but not that of Id3. Note that there is variability in the fold-enrichments in the three experiments: as this is high (from 60 to 800-folds), we consider quantitative changes difficult to interpret, especially when compared to completely negative promoters such as MyoD and Id3. Therefore, we conclude that NF-Y does not regulate MyoD directly—and despite promoter binding—NF-Y has modest effects on Id1 transcription in C2C12 cells.

## 4. Discussion

By genome editing, we derived clones of C2C12 cells that express NF-YAs instead of NF-YAl. We verified that NF-YAs—and companion HFDs—are expressed at comparable levels and that it decreases after differentiation. The edited C2C12 clones are stable, grow normally, yet they are completely deficient in differentiation. We report defects of basal and induced expression of Myomaker and Myomixer, early response-genes likely responsible for lack of cell fusion. Their promoters are targeted by MyoD and Myogenin. In turn, we find low—basal and induced—levels of MyoD and Myogenin in the NF-YAs-expressing clones. Finally, expression of both MRFs are indirectly controlled by NF-Y.

### 4.1. Role of NF-YA Alternative Splicing in Muscle Cells

Specific isoforms of TFs have long been known to impact heavily on transcriptional regulation. Paradigmatic examples are the members of the p53/p63/p73 families, whose isoforms, produced by multiple promoters and alternative splicing, have different targets and often opposing transcriptional effects [55]. The muscle system is no exception [56,57]. Mef2C and Mef2D undergo alternative splicing during muscle differentiation [57,58]: a muscle-specific isoform of Mef2D contains exon α2 rather than α1, both expressed in muscle cells. Growing and early differentiating cells harbors MEF2Dα1; the switch to MEF2Dα2 occurs in terminal stages of C2C12 differentiation, leading to activation of late genes. MEF2Dα1 is phosphorylated at two serines by PKA [59], which mediate association with HDACs, resulting in repression. MEF2Dα2 lacks these residues, functioning as a transcriptional activator. Parallel molecular mechanisms appear to be operating for the related MEF2Cα1/α2 alternative splicing isoforms [58]. The key issue in Mef2 splicing regulation is involvement in late stages of differentiation. Alternative splicing was reported for the master TFs of muscle commitment PAX3 and PAX7, but the functional roles of the single isoforms are less well characterized [60,61,62,63,64].

We show here that a switch from NF-YAl to NF-YAs causes a major difference in the differentiation properties of C2C12 cells. The major NF-YA isoforms, originally reported decades ago [32], are only recently attracting the attention they deserve. In part, this was due to the elusive logic of their expression patterns: in some systems, cells have NF-YAs before—and NF-YAl after—differentiation; in others, such as in muscle cells, NF-YAl is mostly found. In part, it was because of the rather unimpressive nature of the exon 3 amino acids incorporated into NF-YAl: a short stretch rich in glutamines and hydrophobic residues amid the larger transactivation domain. Overexpression experiments suggested differences in gene activation [39,65], but these experiments are to be taken with a grain of salt, because of the large amount of proteins produced, targeting the large number of potential NF-Y sites in the genome. NF-YA AS is likely more complex than what is shown here. First, NF-YAx is another alternatively spliced isoform, recently reported in glioblastomas, devoid of exons 3 and 5: this greatly reduces the activation domain, with important functional consequences [66]. Expression of NF-YAx will have to be monitored in normal cells, to verify whether it is specific for glioblastomas. Second, there are micro differences—6 amino acids—produced in many cell types within the acceptor site of exon 5. Third, some cells show the inclusion of an additional Gln residue at the acceptor splicing site of exon 3, producing a 29 amino acids insertion [32]. Note that a similar situation was reported for PAX3, in which an extra Gln causes differences in DNA-binding affinity [59]. Precise editing techniques, as we have started to use here in C2C12 cells, could sort out the functionality of the various isoforms.

### 4.2. NF-Y Does Not Target Directly Genes Involved in C2C12 Differentiation

Sequence-specific TFs target specific genomic sites, driven by the discriminatory power of their DNA-binding Domains. However, they are also known to be binding indirectly, being tethered by other TFs or complexes: analysis of genomic locations by ENCODE has shown that this latter mechanism is far from marginal [67]. In addition to ENCODE, several independent ChIP-seq of TFs—and cofactors—identified binding to CCAAT locations [68]. One such example regards the orphan receptor Rev-Erb, important for muscle regeneration, targeting NF-Y sites in C2C12 cells [69]. The reverse, namely NF-Y being tethered to CCAAT-less locations by other TFs, has yet to be described. The issue could theoretically be relevant, since the genes down-regulated after NF-Y removal, or by switching from NF-YAl to NF-YAs, have generally no CCCAT in promoters. The effects appear to be largely indirect, but we do not favor the promoter tethering hypothesis. Rather, we report binding of Myogenin and MyoD to the promoters of Myomaker and Myomixer and show that Myogenin overexpression leads to recovery of their expression in C2-YAl-KO cells. This extends to mouse cells genetic experiments made in zebrafish [53]. It also indicates that NF-Y does not regulate other TFs essential for expression of these two genes. In summary, NF-Y/CCAAT interactions in promoters, which are structurally identical for NF-YAl and NF-YAs, are likely not crucial for genes induced during myotubes formation: rather, the focus is shifted to the control of MRFs, or other TFs.

**Figure 7:**
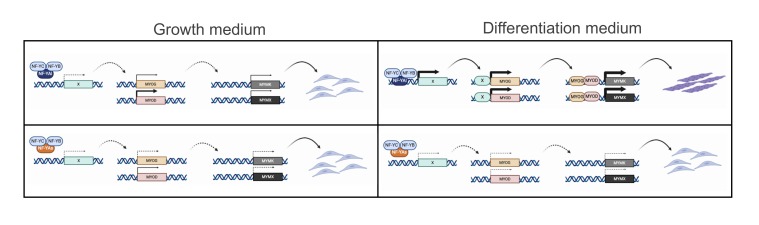
NF-YA isoforms involvement in regulation of expression of muscle genes. Model for NF-YA isoforms mediated regulation of expression of muscle genes in growth condition (left panel) and differentiation condition (right panel).

We have analyzed expression of TFs involved in myoblast/C2C12 differentiation. The majority are not dramatically altered in edited clones. Mef2C induction is impaired, but previous studies indicated that NF-Y is bound to the Mef2D, not to Mef2C promoter [39]. We find that Mef2C, not Mef2D, is regulated by NF-YB RNAi interference. Note that these TFs are also targeted by MyoD and Myogenin, as they play a role in the final stages of differentiation [7,59]. This suggests indirect regulation by NF-Y via MRFs. Id1/Id3 do have bona fide functional CCAAT in promoters [70], bound in cancer cells as per ENCODE data (M. Ronzio, A.B., D.D., R.M., in preparation) and in NTera2 cells [71]: Id1, but not Id3, is bound in vivo by NF-Y in C2C12, parental cells and edited clones. The levels are decreased in C2-YAl-KO upon differentiation, but NF-Y-inactivation brings very marginal decrease in Id1 expression. PAX3, which acts upstream of MyoD, shows variable, somewhat increased mRNA levels in the edited clones, but this is not supported by analysis of protein levels. In summary, there is no clear CCAAT-driven TF that could explain the phenotype: instead, we propose that the decrease of Myogenin and MyoD expression entails a cascade of transcriptional events leading to failure of differentiation (Figure 7).

### 4.3. NF-Y Regulates MRFs Expression Indirectly

Switching from NF-YAl to NF-YAs—and NF-YB inactivation—negatively affects MRFs expression. Myf5 is moderately down in growing cells, remaining somewhat higher after differentiation. NF-Y-inactivation leads to a severe drop in Myogenin expression and a decrease of MyoD, which indicates an impact of NF-Y on their expression. The regulation appears to be transcriptional for Myogenin, not for MyoD, whose mRNA levels are variable, but overall similar. The Myogenin promoter is CCAAT-less and an indirect effect of NF-Y must be invoked. As for MyoD, the promoter harbors a high affinity NF-Y site, extremely conserved in evolution [54] and at the expected position (at -70 from TSS). Yet, NF-Y is not bound in vivo (Figure 6C). This is the only such example in nearly 200 promoters for which genetic analysis was reported [72]. The combination of an evolutionarily conserved, canonical CCAAT in a standard promoter position might function through NF-Y somewhen during the physiological activation of MyoD in development, while it has become expendable in the C2C12 system. Thus, down-regulation of MyoD in NF-YAs-expressing cells is also an indirect effect. It was proposed that MyoD serves as “pioneer” TF predisposing chromatin configurations for Myogenin to act as powerful activator of terminal differentiation genes and repressor of cell-cycle genes [73]. The latter function might be robustly counteracted by NF-YAs, but we have no evidence of that (Figure 2). It is now clear that the focus is set on transcriptional regulation of the MyoD and Myogenin units and on which activator TF(s)—or cofactor(s)—is under NF-YAl—but not NF-YAs—direct control. For the time being, the “candidate” TFs approach used here failed to offer a plausible explanation on how NF-YAl regulates MRFs expression, thereby muscle differentiation. We must resolve to more systematic analysis, such as RNA-seq, to identify potential NF-Y-mediated regulators in C2C12. In light of the low intrinsic levels of muscle-commitment by MRFs in C2-YAl-KO clones, such analysis could also shed light on the actual identity of these cells.

## Supplementary Materials

The following are available online at https://www.mdpi.com/2073-4409/9/3/789/s1, Figure S1: CRISPR/Cas9n system and ablation of NF-YA exon 3 in C2C12 cells. (A) Schematic representation of plasmids construction, following the Multiplex CRISPR/Cas9n Assembly System Kit protocol (Yamamoto lab) [40]. (B) Sequencing of the two C2-YAl-KO clones (#83, #117) compared to the control (ctr). Deleted sequence, targeted sequence and exon 3 sequence are highlighted. Figure S2: Cell-cycle analysis of C2-YAl-KO clones. Flow cytometry analysis of ctr C2C12 cells and the two C2-YAl-KO clones in GM condition. The analysis of three independent experiments and the average of percentage of cells in each cell-cycle phase are shown. Figure S3: Gene expression analysis of TFs in growing and differentiated C2C12 cells. Gene expression analysis by RT-qPCR of key muscle differentiation regulators (left panel) and other TFs shown to be important for muscle differentiation (right panel) in GM condition and 24 h after differentiation in C2C12 ctr cells. Error bars represent the SD of three different experiments. P-values were calculated using the one-sample t-test. Figure S4: Myomaker and Myomixer expression are regulated by MyoD and Myogenin. (A) UCSC view of Mymk and Mymk *loci* showing alignment of ChIP-Seq data and DNA regulatory motifs conserved across Vertebrates by PhastCons. (B) Phase-contrast analysis of C2C12 cells (ctr and #83) morphology transfected with pEmpty or pMyog, 96 h after differentiation. Figure S5: Analysis of NF-Y involvement in muscle specific genes expression. (A) Gene expression analysis by RT-qPCR of NF-YB and key muscle differentiation regulators in C2C12 cells 72 h after NF-YB silencing (siNF-YB) and the scrambled siRNA control (II° experiment). Error bars represent the SD of two different q-PCR replicates. *p*-values were calculated using the one-sample t-test. (B) Western blot analysis of NF-YB, NF-YA and key muscle differentiation regulators (Myogenin, MyoD) protein levels 72 h after NF-YB silencing (siNF-YB) and the scrambled control. Vinculin was used as loading control. (C) Analysis of II° and III° ChIP experiments performed on C2C12 ctr cells in GM condition using NF-YB antibody and the negative control (nc). The unrelated region (ur) and Stard4 were used as negative and positive control, respectively. Results are represented as the input percentage of sample normalized to the nc. Table S1: Off-targets analysis. Analysis of possible off-target sites of each gRNA using the online tool https://crispr.cos.uni-heidelberg.de. For each gRNA the off-target gene name, gene id, position (intronic, intergenic, exonic), mismatches (MM) and the PAM sequence are reported. Table S2: Primers used. The specific sequence of each primer (forward and reverse) used for RT-qPCR and ChIP analysis are reported.

## References

[1] Buckingham M. (2001). Skeletal muscle formation in vertebrates. Curr. Opin. Genet. Dev..

[2] Buckingham M., Rigby P.W. (2014). Gene Regulatory Networks and Transcriptional Mechanisms that Control Myogenesis. Dev. Cell.

[3] Hernández-Hernández J.M., García-González E.G., Brun C.E., Rudnicki M.A. (2017). The myogenic regulatory factors, determinants of muscle development, cell identity and regeneration. Semin. Cell Dev. Boil..

[4] Zammit P.S. (2017). Function of the myogenic regulatory factors Myf5, MyoD, Myogenin and MRF4 in skeletal muscle, satellite cells and regenerative myogenesis. Semin. Cell Dev. Boil..

[5] Buckingham M., Relaix F. (2015). PAX3 and PAX7 as upstream regulators of myogenesis. Semin. Cell Dev. Boil..

[6] Black B.L., Olson E.N. (1998). Transcriptional Control Of Muscle Development by Myocyte Enhancer Factor-2 (MEF2) Proteins. Annu. Rev. Cell Dev. Boil..

[7] Taylor M.V., Hughes S.M. (2017). Mef2 and the skeletal muscle differentiation program. Semin. Cell Dev. Boil..

[8] Kumar D., Shadrach J.L., Wagers A.J., Lassar A.B. (2009). Id3 Is a Direct Transcriptional Target of Pax7 in Quiescent Satellite Cells. Mol. Boil. Cell.

[9] Wu J., Lim R.W. (2005). Regulation of inhibitor of differentiation gene 3 (Id3) expression by Sp2-motif binding factor in myogenic C2C12 cells: Downregulation of DNA binding activity following skeletal muscle differentiation. Biochim. et Biophys. Acta (BBA) Gene Struct. Expr..

[10] Atherton G.T., Travers H., Deed R., Norton J.D. (1996). Regulation of cell differentiation in C2C12 myoblasts by the Id3 helix-loop-helix protein. Cell Growth Differ. Mol. Boil. J. Am. Assoc. Cancer Res..

[11] Soleimani V.D., Yin H., Jahani-Asl A., Ming H., Kockx C., Van Ijcken W.F.J., Grosveld F., Rudnicki M.A. (2012). Snail regulates MyoD binding-site occupancy to direct enhancer switching and differentiation-specific transcription in myogenesis. Mol. Cell.

[12] Grifone R., Demignon J., Houbron C., Souil E., Niro C., Seller M.J., Hamard G., Maire P. (2005). Six1 and Six4 homeoproteins are required for Pax3 and Mrf expression during myogenesis in the mouse embryo. Dev..

[13] Yajima H., Motohashi N., Ono Y., Sato S., Ikeda K., Masuda S., Yada E., Kanesaki H., Miyagoe-Suzuki Y., Takeda S. (2010). Six family genes control the proliferation and differentiation of muscle satellite cells. Exp. Cell Res..

[14] Santolini M., Sakakibara I., Gauthier M., Aulinas F.R., Takahashi H., Sawasaki T., Mouly V., Concordet J.-P., Defossez P.-A., Hakim V. (2016). MyoD reprogramming requires Six1 and Six4 homeoproteins: genome-wide cis-regulatory module analysis. Nucleic Acids Res..

[15] Yajima H., Kawakami K. (2016). Low Six4 and Six5 gene dosage improves dystrophic phenotype and prolongs life span of mdx mice. Dev. Growth Differ..

[16] Guadagnin E., Mázala D., Chen Y.-W. (2018). STAT3 in Skeletal Muscle Function and Disorders. Int. J. Mol. Sci..

[17] Messina G., Biressi S.A.M., Monteverde S., Magli A., Cassano M., Perani L., Roncaglia E., Tagliafico E., Starnes L., Campbell C.E. (2010). Nfix Regulates Fetal-Specific Transcription in Developing Skeletal Muscle. Cell.

[18] Rossi G., Antonini S., Bonfanti C., Monteverde S., Vezzali C., Tajbakhsh S., Cossu G., Messina G. (2016). Nfix Regulates Temporal Progression of Muscle Regeneration through Modulation of Myostatin Expression. Cell Rep..

[19] Hayashi S., Manabe I., Suzuki Y., Relaix F., Oishi Y. (2016). Klf5 regulates muscle differentiation by directly targeting muscle-specific genes in cooperation with MyoD in mice. eLife.

[20] Sunadome K., Yamamoto T., Ebisuya M., Kondoh K., Sehara-Fujisawa A., Nishida E. (2011). ERK5 Regulates Muscle Cell Fusion through Klf Transcription Factors. Dev. Cell.

[21] Potthoff M.J., Olson E.N. (2007). MEF2: a central regulator of diverse developmental programs. Dev..

[22] Ling F., Kang B., Sun X.H. (2014). Id proteins: small molecules, mighty regulators. Curr. Top Dev. Biol..

[23] Christensen K.L., Patrick A.N., McCoy E.L., Ford H.L. (2008). Chapter 5 The Six Family of Homeobox Genes in Development and Cancer. Advances in Cancer Research.

[24] Bialkowska A., Yang V.W., Mallipattu S.K. (2017). Krüppel-like factors in mammalian stem cells and development. Dev..

[25] Piper M., Gronostajski R., Messina G. (2019). Nuclear Factor One X in Development and Disease. Trends Cell Boil..

[26] Dolfini D., Gatta R., Mantovani R. (2011). NF-Y and the transcriptional activation of CCAAT promoters. Crit. Rev. Biochem. Mol. Boil..

[27] Fleming J.D., Pavesi G., Benatti P., Imbriano C., Mantovani R., Struhl K. (2013). NF-Y coassociates with FOS at promoters, enhancers, repetitive elements, and inactive chromatin regions, and is stereo-positioned with growth-controlling transcription factors. Genome Res..

[28] Sherwood R.I., Hashimoto T., O’Donnell C.P., Lewis S., A Barkal A., Van Hoff J.P., Karun V., Jaakkola T., Gifford D.K. (2014). Discovery of directional and nondirectional pioneer transcription factors by modeling DNase profile magnitude and shape. Nat. Biotechnol..

[29] Oldfield A., Yang P., Conway A.E., Cinghu S., Freudenberg J., Yellaboina S., Jothi R. (2014). Histone-fold domain protein NF-Y promotes chromatin accessibility for cell type-specific master transcription factors. Mol. Cell.

[30] Oldfield A., Henriques T., Kumar D., Burkholder A.B., Cinghu S., Paulet D., Bennett B.D., Yang P., Scruggs B.S., Lavender C.A. (2019). NF-Y controls fidelity of transcription initiation at gene promoters through maintenance of the nucleosome-depleted region. Nat. Commun..

[31] Lu F., Liu Y., Inoue A., Suzuki T., Zhao K., Zhang Y. (2016). Establishing Chromatin Regulatory Landscape during Mouse Preimplantation Development. Cell.

[32] Li X.Y., Van Huijsduijnen R.H., Mantovani R., Benoist C., Mathis D. (1992). Intron-exon organization of the NF-Y genes. Tissue-specific splicing modifies an activation domain. J. Boil. Chem..

[33] Ceribelli M., Benatti P., Imbriano C., Mantovani R. (2009). NF-YC Complexity Is Generated by Dual Promoters and Alternative Splicing. J. Biol. Chem..

[34] Benatti P., Dolfini D., Vigano M.A., Ravo M., Weisz A., Imbriano C. (2011). Specific inhibition of NF-Y subunits triggers different cell proliferation defects. Nucleic Acids Res..

[35] Farina A., Manni I., Fontemaggi G., Tiainen M., Cenciarelli C., Bellorini M., Mantovani R., Sacchi A., Piaggio G. (1999). Down-regulation of cyclin B1 gene transcription in terminally differentiated skeletal muscle cells is associated with loss of functional CCAAT-binding NF-Y complex. Oncogene.

[36] Gurtner A., Manni I., Fuschi P., Mantovani R., Guadagni F., Sacchi A., Piaggio G. (2003). Requirement for Down-Regulation of the CCAAT-binding Activity of the NF-Y Transcription Factor during Skeletal Muscle Differentiation. Mol. Boil. Cell.

[37] Gurtner A., Fuschi P., Magi F., Colussi C., Gaetano C., Dobbelstein M., Sacchi A., Piaggio G. (2008). NF-Y Dependent Epigenetic Modifications Discriminate between Proliferating and Postmitotic Tissue. PLOS ONE.

[38] Goeman F., Manni I., Artuso S., Ramachandran B., Toietta G., Bossi G., Rando G., Cencioni C., Germoni S., Straino S. (2012). Molecular imaging of nuclear factor-Y transcriptional activity maps proliferation sites in live animals. Mol. Boil. Cell.

[39] Basile V., Baruffaldi F., Dolfini D., Belluti S., Benatti P., Ricci L., Artusi V., Tagliafico E., Mantovani R., Molinari S. (2016). NF-YA splice variants have different roles on muscle differentiation. Biochim. et Biophys. Acta (BBA) - Gene Regul. Mech..

[40] Mauro A. (1961). Satellite cell of skeletal muscle fibers. J. Cell Boil..

[41] Maity S.N. (2016). NF-Y (CBF) regulation in specific cell types and mouse models. Biochim. et Biophys. Acta (BBA) Bioenerg..

[42] Sakuma T., Nishikawa A., Kume S., Chayama K., Yamamoto T. (2014). Multiplex genome engineering in human cells using all-in-one CRISPR/Cas9 vector system. Sci. Rep..

[43] Dolfini D., Minuzzo M., Pavesi G., Mantovani R. (2012). The Short Isoform of NF-YA Belongs to the Embryonic Stem Cell Transcription Factor Circuitry. STEM CELLS.

[44] Cullot G., Boutin J., Toutain J., Prat F., Pennamen P., Rooryck C., Teichmann M., Rousseau E., Lamrissi-Garcia I., Guyonnet-Duperat V. (2019). CRISPR-Cas9 genome editing induces megabase-scale chromosomal truncations. Nat. Commun..

[45] Min Y.-L., Bassel-Duby R., Olson E.N. (2018). CRISPR Correction of Duchenne Muscular Dystrophy. Annu. Rev. Med..

[46] Bungartz G., Land H., Scadden D.T., Emerson S.G. (2012). NF-Y is necessary for hematopoietic stem cell proliferation and survival. Blood.

[47] Belluti S., Semeghini V., Basile V., Rigillo G., Salsi V., Genovese F., Dolfini D., Imbriano C. (2018). An autoregulatory loop controls the expression of the transcription factor NF-Y. Biochim. et Biophys. Acta (BBA) Bioenerg..

[48] Moran J., Li Y., Hill A.A., Mounts W.M., Miller C.P. (2002). Gene expression changes during mouse skeletal myoblast differentiation revealed by transcriptional profiling. Physiol. Genom..

[49] Clever J.L., Sakai Y., Wang R.A., Schneider D.B. (2010). Inefficient skeletal muscle repair in inhibitor of differentiation knockout mice suggests a crucial role for BMP signaling during adult muscle regeneration. Am. J. Physiol. Physiol..

[50] Salizzato V., Zanin S., Borgo C., Lidron E., Salvi M., Rizzuto R., Pallafacchina G., Donella-Deana A. (2019). Protein kinase CK2 subunits exert specific and coordinated functions in skeletal muscle differentiation and fusogenic activity. FASEB J..

[51] Millay D.P., Gamage D.G., Quinn M.E., Min Y.-L., Mitani Y., Bassel-Duby R., Olson E.N. (2016). Structure–function analysis of myomaker domains required for myoblast fusion. Proc. Natl. Acad. Sci..

[52] Petrany M.J., Millay D.P. (2019). Cell Fusion: Merging Membranes and Making Muscle. Trends Cell Boil..

[53] Ganassi M., Badodi S., Quiroga H.P.O., Zammit P.S., Hinits Y., Hughes S.M. (2018). Myogenin promotes myocyte fusion to balance fibre number and size. Nat. Commun..

[54] Pedraza-Alva G., Zingg J.M., Jost J.P. (1994). AP-1 binds to a putative cAMP response element of the MyoD1 promoter and negatively modulates MyoD1 expression in dividing myoblasts. J. Boil. Chem..

[55] Murray-Zmijewski F., Lane D.P., Bourdon J.C. (2006). p53/p63/p73 isoforms: an orchestra of isoforms to harmonise cell differentiation and response to stress. Cell Death Differ..

[56] Imbriano C., Molinari S. (2018). Alternative Splicing of Transcription Factors Genes in Muscle Physiology and Pathology. Genes.

[57] Nakka K., Ghigna C., Gabellini D., Dilworth F.J. (2018). Diversification of the muscle proteome through alternative splicing. Skelet. Muscle.

[58] Zhang M., Zhu B., Davie J. (2014). Alternative Splicing of MEF2C pre-mRNA Controls Its Activity in Normal Myogenesis and Promotes Tumorigenicity in Rhabdomyosarcoma Cells*. J. Boil. Chem..

[59] Sebastian S., Faralli H., Yao Z., Rakopoulos P., Palii C., Cao Y., Singh K., Liu Q.-C., Chu A., Aziz A. (2013). Tissue-specific splicing of a ubiquitously expressed transcription factor is essential for muscle differentiation. Genome Res..

[60] Vogan K., Underhill D.A., Gros P. (1996). An alternative splicing event in the Pax-3 paired domain identifies the linker region as a key determinant of paired domain DNA-binding activity. Mol. Cell. Boil..

[61] Barber T.D., Barber M.C., Cloutier T.E., Friedman T.B. (1999). PAX3 gene structure, alternative splicing and evolution. Gene.

[62] Pritchard C., Grosveld G., Hollenbach A.D. (2003). Alternative splicing of Pax3 produces a transcriptionally inactive protein. Gene.

[63] Charytonowicz E., Matushansky I., Castillo-Martin M., Hricik T., Cordon-Cardo C., Ziman M. (2011). Alternate PAX3 and PAX7 C-terminal isoforms in myogenic differentiation and sarcomagenesis. Clin. Transl. Oncol..

[64] Vorobyov E., Horst J. (2004). Expression of two protein isoforms of PAX7 is controlled by competing cleavage-polyadenylation and splicing. Gene.

[65] LiBetti D., Bernardini A., Chiaramonte M.L., Minuzzo M., Gnesutta N., Messina G., Dolfini D., Mantovani R. (2019). NF-YA enters cells through cell penetrating peptides. Biochim. et Biophys. Acta (BBA) Bioenerg..

[66] Cappabianca L., Farina A.R., Di Marcotullio L., Infante P., De Simone D., Sebastiano M., Mackay A. (2019). Discovery, characterization and potential roles of a novel NF-YAx splice variant in human neuroblastoma. J. Exp. Clin. Cancer Res..

[67] Wang J., Zhuang J., Iyer S., Lin X., Whitfield T.W., Greven M.C., Pierce B.G., Dong X., Kundaje A., Cheng Y. (2012). Sequence features and chromatin structure around the genomic regions bound by 119 human transcription factors. Genome Res..

[68] Zambelli F., Pavesi G. (2017). Genome wide features, distribution and correlations of NF-Y binding sites. Biochim. et Biophys. Acta (BBA) Bioenerg..

[69] Welch R.D., Guo C., Sengupta M., Carpenter K.J., Stephens N.A., Arnett S.A., Meyers M.J., Sparks L.M., Smith S.R., Zhang J. (2017). Rev-Erb co-regulates muscle regeneration via tethered interaction with the NF-Y cistrome. Mol. Metab..

[70] Van Wageningen S., Ridder M.C.B.-D., Nigten J., Nikoloski G., Erpelinck-Verschueren C.A.J., Löwenberg B., De Witte T., Tenen D.G., Van Der Reijden B.A., Jansen J.H. (2008). Gene transactivation without direct DNA binding defines a novel gain-of-function for PML-RARα. Blood.

[71] Moeinvaziri F., Shahhosseini M. (2015). Epigenetic role of CCAAT box-binding transcription factor NF-Y onIDgene family in human embryonic carcinoma cells. IUBMB Life.

[72] Dolfini D., Mantovani R., Zambelli F., Pavesi G. (2009). A perspective of promoter architecture from the CCAAT box. Cell Cycle.

[73] Singh K., Dilworth F.J. (2013). Differential modulation of cell cycle progression distinguishes members of the myogenic regulatory factor family of transcription factors. FEBS J..

