# Supplementary figures and images for: The Switch from NF-YAl to NF-YAs Isoform Impairs Myotubes Formation
Source: Cells. 2020 Mar 24;9(3):789. doi: 10.3390/cells9030789 (PMC7140862; doi:10.3390/cells9030789)

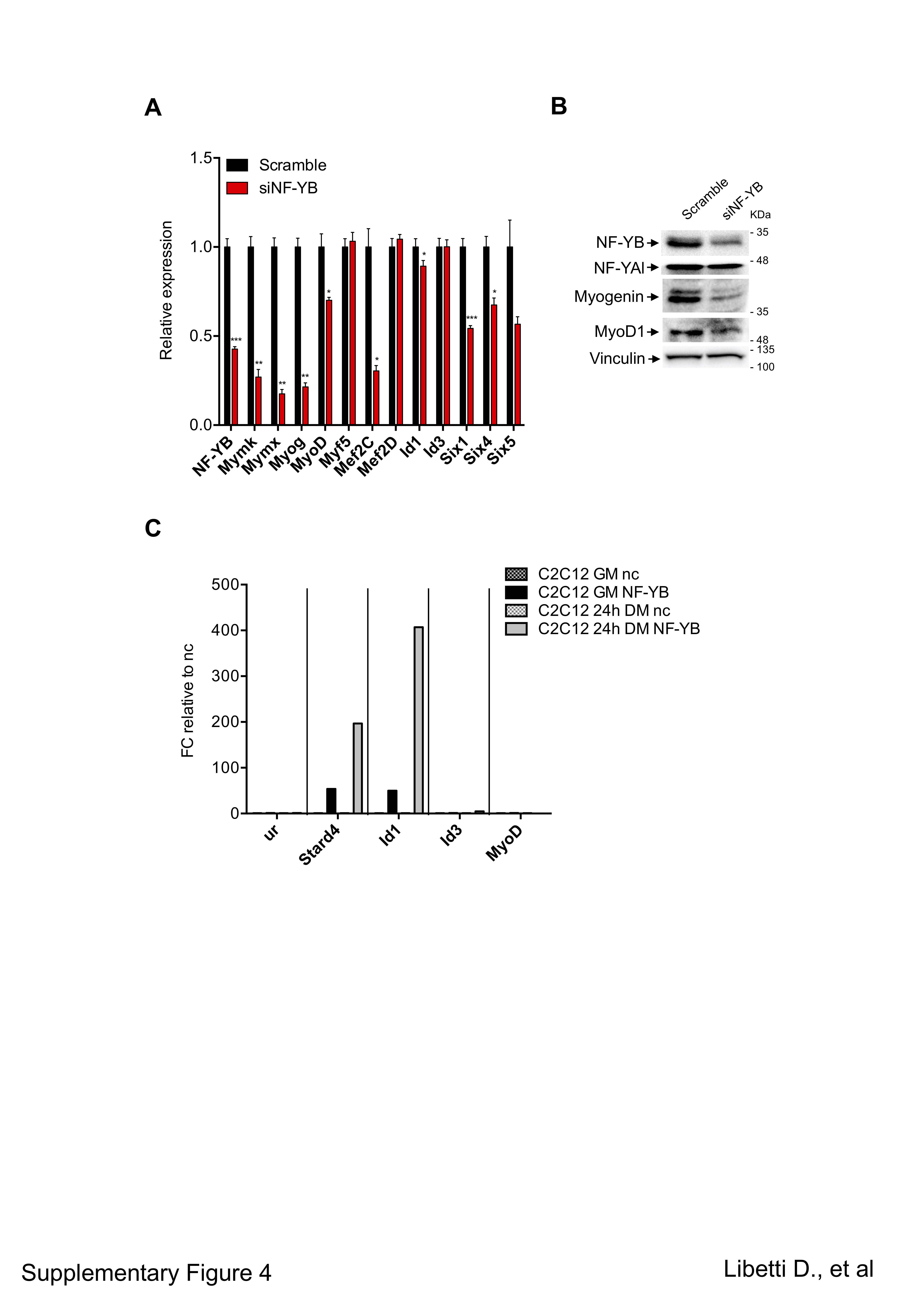

Supplement: Supplementary file 1 [file cells-09-00789-s001.zip › Suppl. Figure Singole JPEG/Suppl. Figure 4.jpg]

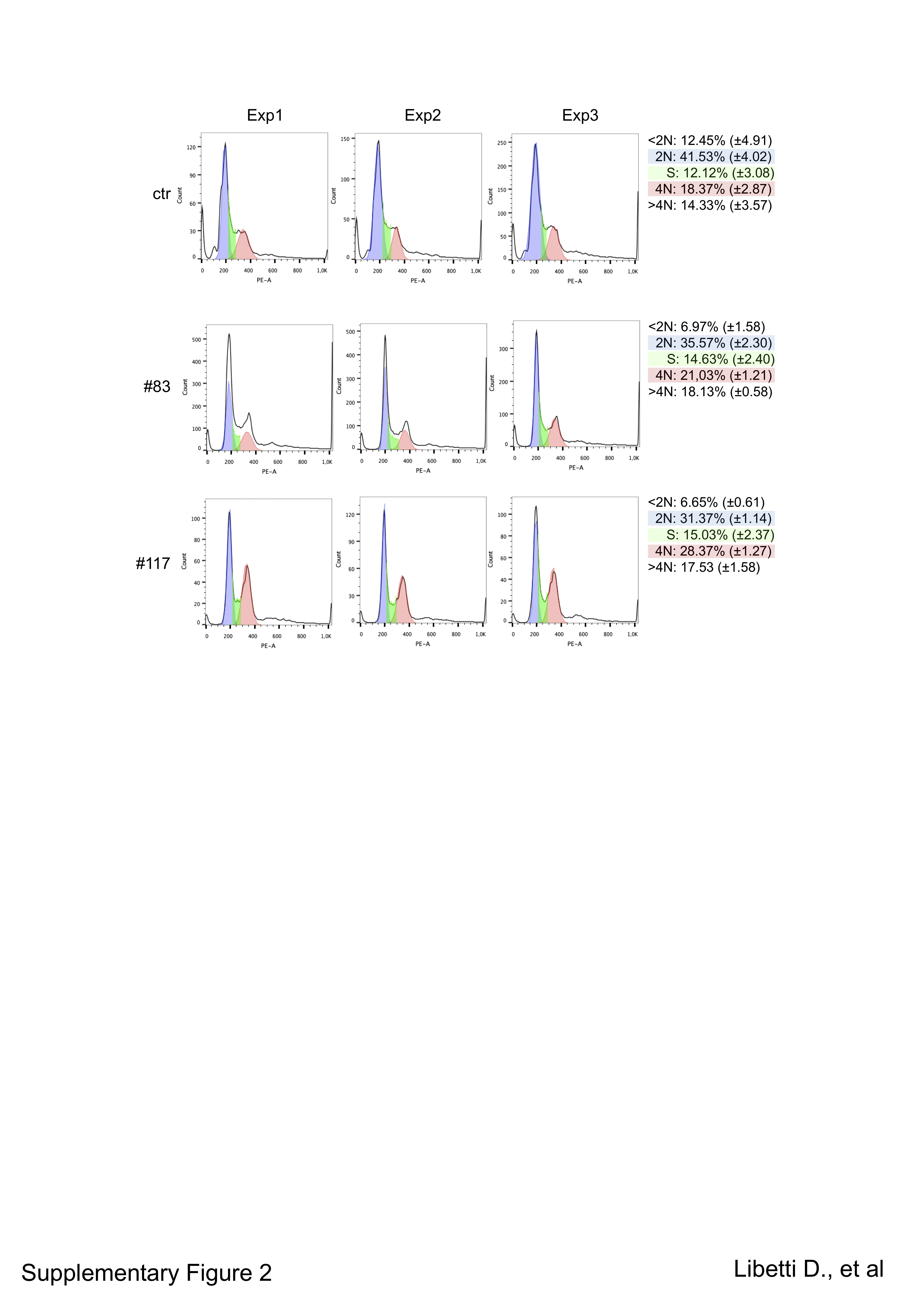

Supplement: Supplementary file 1 [file cells-09-00789-s001.zip › Suppl. Figure Singole JPEG/Suppl. Figure 2.jpg]

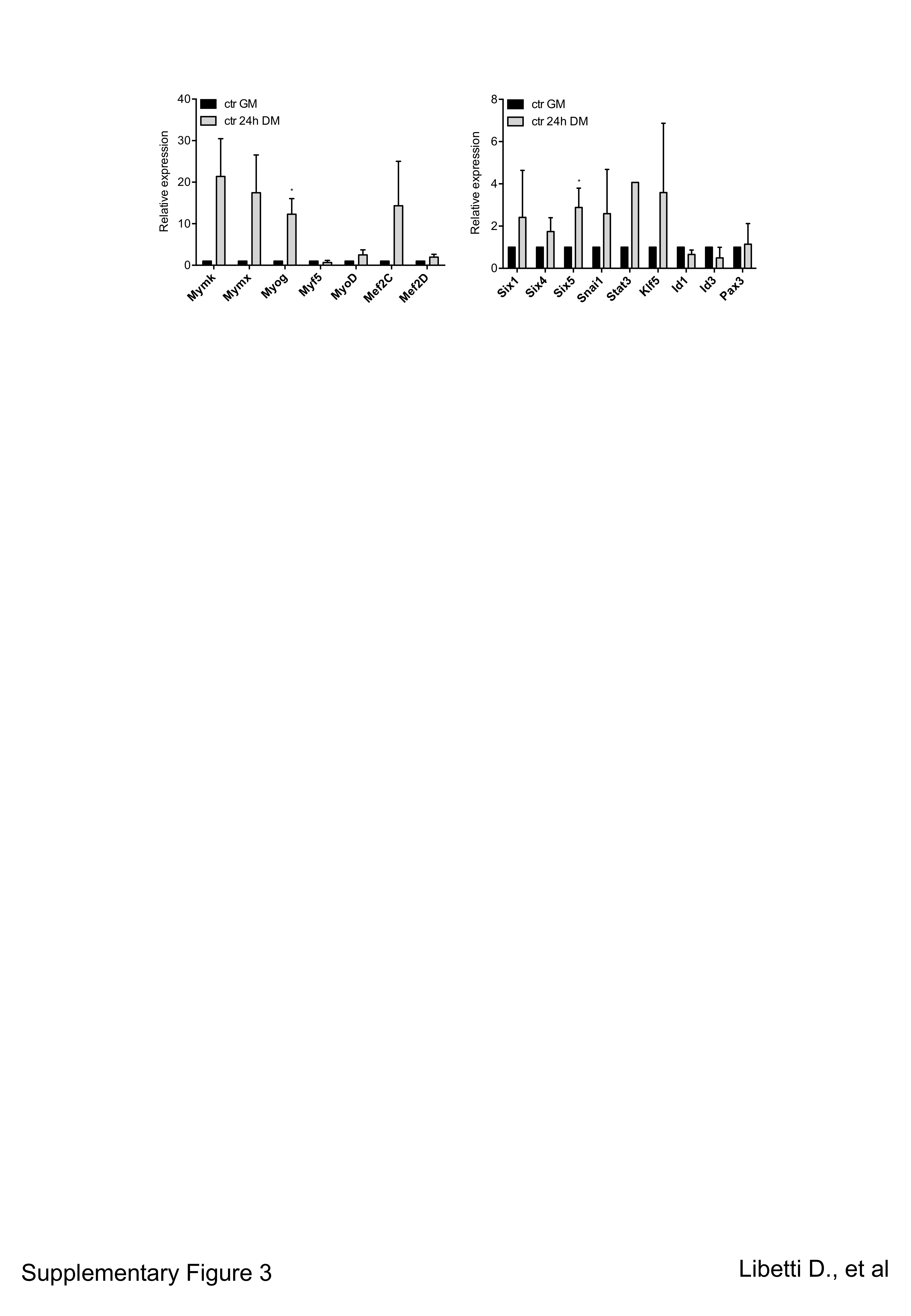

Supplement: Supplementary file 1 [file cells-09-00789-s001.zip › Suppl. Figure Singole JPEG/Suppl. Figure 3.jpg]

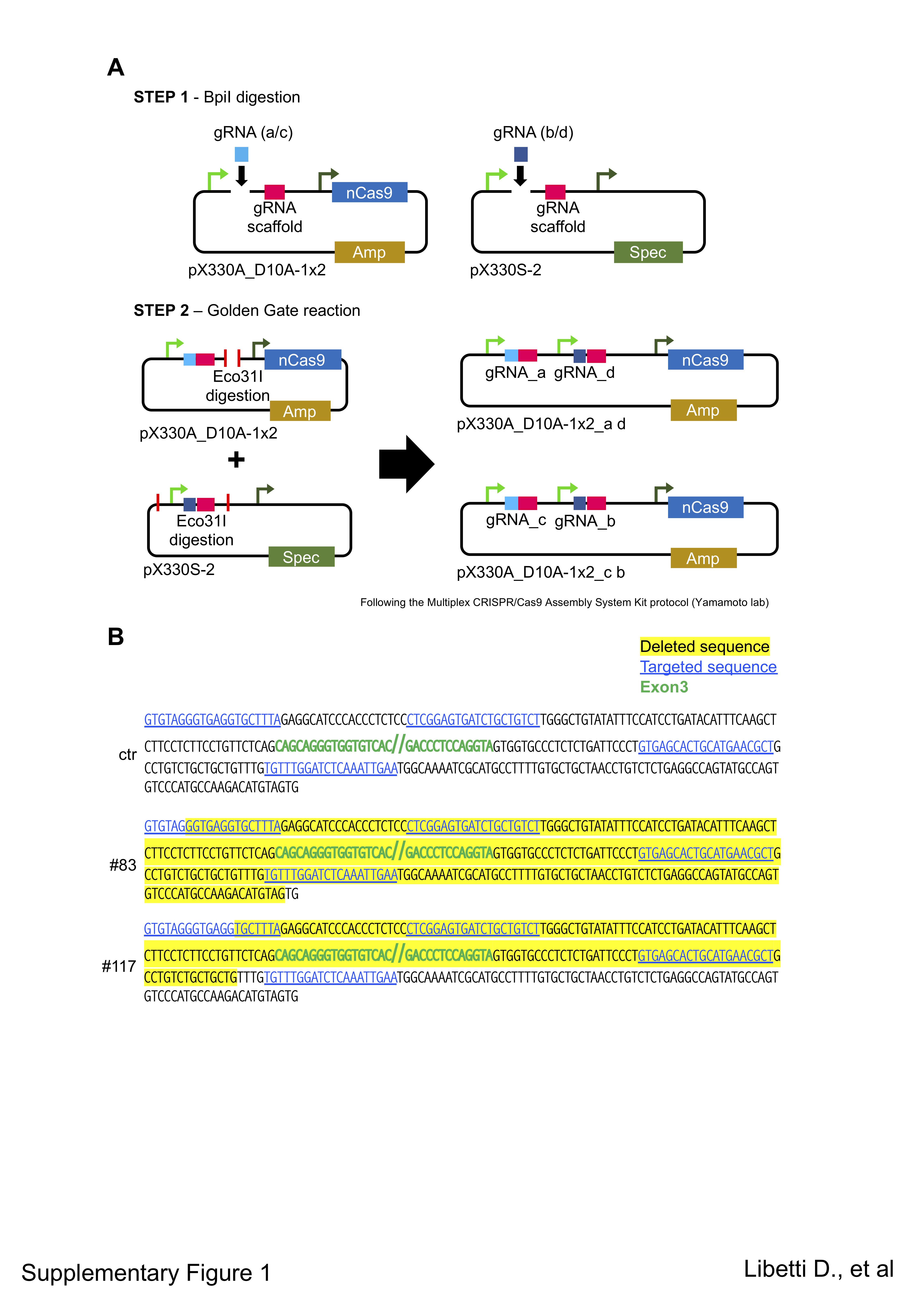

Supplement: Supplementary file 1 [file cells-09-00789-s001.zip › Suppl. Figure Singole JPEG/Suppl. Figure 1.jpg]
